# Machine learning algorithms enhance the accuracy of radiographic diagnosis of dental caries: a comparative study

**DOI:** 10.1093/dmfr/twaf053

**Published:** 2025-07-10

**Authors:** Shwetha Hegde, Jinlong Gao, Stephen Cox, Shanika Nanayakkara, Rena Logothetis, Rajesh Vasa

**Affiliations:** Dentomaxillofacial Radiology, Sydney Dental School, University of Sydney, Sydney, 2010, Australia; Sydney Dental School, Institute of Dental Research, Westmead Centre for Oral Health, University of Sydney, Sydney, 2145, Australia; Discipline of Oral Surgery, Sydney Dental School, University of Sydney, Sydney, 2010, Australia; Sydney Dental School, Institute of Dental Research, Westmead Centre for Oral Health, University of Sydney, Sydney, 2145, Australia; Applied Artificial Intelligence Institute, Deakin University, Melbourne, 3125, Australia; Translational Research and Development, Applied Artificial Intelligence Institute, Deakin University, Melbourne, 3125, Australia

**Keywords:** dental caries diagnosis, bitewing radiography, cognitive aids, machine learning, checklists

## Abstract

**Objectives:**

This study evaluated the influence of cognitive aids, including machine learning (ML) algorithms and checklists, on the diagnostic accuracy and confidence of dental students in detecting dental caries on bitewing radiographs.

**Methods:**

Fifty-two third-year dental students were randomly assigned to control, ML, or checklist groups. The participants recorded their caries diagnoses (charting) on 10 bitewing radiographs and rated their confidence. Diagnostic accuracy and reliability were compared between groups for caries detection (present/absent). The inter-rater reliability for International Caries Detection and Assessment System II (ICDAS II) caries grading was assessed using weighted kappa. Participants also completed questionnaires on their perceptions of cognitive aids.

**Results:**

ML group showed the highest diagnostic accuracy and confidence levels. For caries detection, the ML group achieved the highest sensitivity (79%) and diagnostic odds ratio (20.3), while the checklist group had the highest specificity (90.9%) (*P* < .001). The control group showed moderate sensitivity (67.9%) but outperformed the checklist group in this metric. Inter-rater agreement for caries detection was highest in the ML group (κ = 0.702, 95% CI: 0.692-0.713; *P* < .001), followed by the checklist group. The ML group also had the highest weighted kappa for ICDAS II grading (κ = 0.520, *P* < .001). Confidence levels differed significantly between groups (*P* < .001), with the ML group reporting the highest confidence.

**Conclusion:**

ML algorithms may enhance diagnostic accuracy and confidence, possibly by reducing cognitive load. While standardizing the diagnostic process, checklists were less effective, likely due to their lack of flexibility and clinical context. Further research is needed to better understand our findings and translate them into clinical workflows.

## Introduction

The diagnostic process relies on the dentist’s ability to detect subtle differences between normal and abnormal findings in images and apply their knowledge to make accurate diagnoses.^1^ Accurately detecting abnormalities on radiographs can lead to early detection of conditions and timely interventions, ultimately improving patient outcomes. However, the fast-paced and high-pressure nature of clinical environments increases the likelihood of errors and results in misdiagnosis and inappropriate treatment plans.^2^ This may occur due to cognitive overload that impairs clinical decision-making.^3^^,^^4^

Cognitive load refers to the mental effort needed to complete a task or achieve a learning goal.^5^ The cognitive load theory (CLT) describes how humans process new information by forming cognitive schemas—mental structures that help organize and interpret information—such as those used to systematically analyse a radiographic image.^6^^,^^7^ CLT outlines three key components: (1) memory systems, which include sensory, working and long-term memory; (2) processes involved in learning; and (3) various types of cognitive load that affect working memory.^8^ According to the CLT, working memory is finite and can temporarily hold new information necessary for complex tasks, whereas long-term memory is more stable and organized, storing memory for extended periods and enabling more efficient information processing.^9^ In situations of cognitive overload, the demands of working memory exceed capacity, and clinicians may miss critical information, leading to diagnostic errors.^10^ For example, errors may occur when a clinician is presented with unfamiliar radiographic patterns or when the radiographic findings do not correlate with the clinical information.^11^ These errors are relevant in dentistry, where accurate interpretation of dental radiographs is essential for diagnosing conditions like dental caries.

Dental caries remains a prevalent dental problem globally, and general dentists play a central role in the diagnosis and management of caries.^12^^,^^13^ Diagnosing dental caries relies on clinical and radiographic examinations, and intraoral bitewing radiographs are a valuable diagnostic tool.^14–16^ Studies investigating the diagnostic efficacy of dental imaging techniques in caries diagnosis have reported that intraoral bitewing radiographs are better than periapical, extraoral, and panoramic radiographs.^17^ The radiographic diagnosis of caries is influenced by perceptual and cognitive factors, the clinician’s experience, expertise, and results in variability in the diagnosis of location and extent of caries.^18–21^ The accuracy of caries diagnosis can also be influenced by the number and distribution of carious lesions visible on a radiograph, as previous research has demonstrated that the likelihood of detecting caries increases when more lesions and restorations are already present in the dentition.^22^ False positive and false negative diagnoses and inter-observer and intra-observer variations further affect the diagnostic accuracy of bitewing radiographs.^23^ The variations in radiographic caries diagnosis are also influenced by the location and depth of carious lesions, with higher accuracy observed for diagnosing dentinal caries on interproximal surfaces.^24^ Initial caries on bitewing radiographs have low sensitivity and reasonable specificity, whereas deeper dentinal caries on interproximal surfaces have higher sensitivity and specificity.^25^ These discrepancies in radiographic diagnosis can result in undertreatment or overtreatment of carious lesions.^26–28^

To address these challenges and improve the accuracy of caries diagnosis, various techniques have been developed, including templates, checklists, clinical decision support systems, and machine learning algorithms.^29–31^ These techniques can serve as cognitive aids by simplifying complex tasks, assisting in decision-making by reducing mental effort and facilitating improved decision-making.^32–34^ These cognitive aids have been tested in different clinical settings in medicine and have been shown to reduce errors, increase adherence to best practices and improve overall performance. Research has indicated that using guided checklists increases the accuracy of medical diagnoses and reduces errors in radiographic interpretation. However, their impact on diagnostic accuracy has not been measured quantitatively.^35^^,^^36^ Checklists (CL) are underutilized in dentistry, and to the best of our knowledge, no studies have assessed their influence on the accuracy of radiographic caries diagnosis. In contrast, machine learning (ML) algorithms using deep learning techniques have been used to assist in the radiographic detection of dental caries.^30^^,^^37^^,^^38^ Several studies have shown that algorithms increase diagnostic accuracy and reduce interpretation errors, with diagnostic accuracy between 80% and 90%.^30^^,^^38^

This study aimed to evaluate the influence of cognitive aids on the accuracy of diagnosing caries using bitewing radiographs. Specifically, the study aimed to compare the diagnostic accuracy of 3 groups of dental students: those using no aids, those using a diagnostic checklist, and those assisted by an ML caries diagnosis algorithm. Additionally, the study aimed to evaluate the participant’s self-reported confidence in their caries diagnosis and their opinions and perceptions about ML algorithms and checklists on diagnostic performance.

## Methods

### Study design and participant selection

Dental students in their third academic year in the Doctor of Dental Medicine (DMD) programme at Sydney Dental School, University of Sydney, were invited to participate in this study. The third-year dental students have a sound theoretical understanding of caries identification and the International Caries Detection and Assessment II (ICDAS II)^39^ grading. They began seeing patients in a supervised clinical setting from the second year onward. Their radiology training included approximately 15 h of clinical experience where they actively took and interpreted intraoral and extraoral radiographs, completed structured radiographic assessments using ICDAS II and received feedback from clinical tutors. Additionally, during their clinical sessions in the student clinics, students routinely took and interpreted bitewing radiographs as part of the patient care, and their findings were reviewed by supervising clinicians. These combined experiences ensured that students had the necessary foundational knowledge and practical competence in caries diagnosis to participate in this study. However, no formal calibration for ICDAS II grading was conducted between the students and the clinical tutors specifically for this study. Additionally, while some participants may have had prior clinical experience (eg working as a dental assistant), which could introduce potential bias, this was mitigated in several ways. First, all participants received similar training in radiographic diagnosis of dental caries as a part of the DMD curriculum, ensuring comparable foundational knowledge. Furthermore, random allocation to the study groups helped control for individual differences in prior experience.

By recruiting dental students, who generally have similar levels of clinical experience, clinical experience was excluded as a confounding factor in diagnostic accuracy. This approach ensured that variations in diagnostic performance were not due to differences in clinical experience. This study received ethics approval from the University of Sydney’s Human Research Ethics Committee (approval no. 2023/019). Based on the sample size calculation, each of the 3 groups- control, machine learning (ML), and checklist (CL), was required to have 16 participants. This calculation was made using a confidence interval of 95% and power of 80% with a 1:1:1 group ratio and expected variation in success rates of 60%, 70%, and 80% (standard deviation of 10%).

### Bitewing radiograph selection and quality assessment

For this study, the 10 adult bitewings of the highest technical quality were selected from the private collection of 50 de-identified bitewings belonging to the research team. Bitewing radiographs showing posterior teeth from the distal surfaces of the first premolars to the mesial surfaces of the second molars were considered for quality assessment. Two researchers (SH and JG) conducted a quality assessment (QA) of the images. The inclusion criteria required the radiographs to show adequate brightness, contrast, sharpness, and absence of significant artefacts. The following quality assessment criteria were applied: QA1-technically perfect, QA2-diagnostically acceptable, and QA3-non-diagnostic.^40^ Only images with QA1 and QA2 ratings were included. Those radiographs with cone cuts or overlapping interproximal surfaces were also excluded. Of the 50 images initially reviewed, 40 (80%) were excluded based on these criteria. Bitewings that met the inclusion criteria were allocated a reference number.

### Gold standard consensus

**Figure 1.**
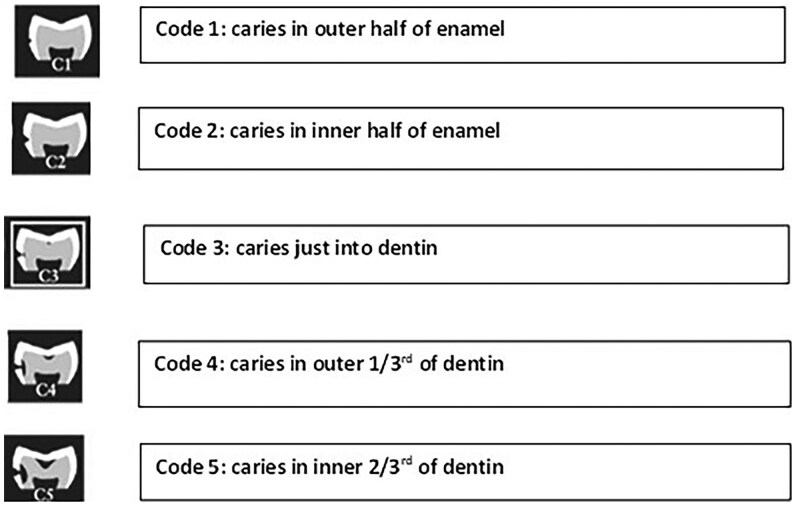
ICDAS II radiographic caries diagnosis criteria.^39^

A panel of 5 experienced clinicians served as the reference standard for diagnosing caries on each bitewing radiograph. The expert panel included 4 experts in dentomaxillofacial radiology with clinical experience ranging from 20 to 50 years, holding positions as faculty members at Sydney Dental School and radiology consultant roles outside of the University. The fifth expert was a general dentist with 35 years of clinical experience. Specifically, each panel member independently reviewed every bitewing and assigned a caries diagnosis for each tooth surface, noting the presence or absence of caries and, where relevant, assigning a score based on the ICDAS II system. The ICDAS II radiographic criteria describe caries severity based on the depth of caries in enamel and dentine (Code 0- no caries; Code 1- caries involved half the thickness of enamel; Code 2- caries involving the entire thickness of enamel; Code 3-caries extending just into dentine [DEJ]; Code 4-caries involved outer 1/3 of dentine; Code 5- caries involving inner 2/3rd of dentine) (Figure 1). Occlusal caries was also included as bitewings, which serve as an important adjunct, although occlusal caries are primarily diagnosed clinically. Bitewings are particularly useful for detecting dentine involvement or lesions not clearly visible on clinical examination alone.

Areas of disagreement were identified by comparing the expert’s diagnosis, and a consensus meeting was held to resolve these discrepancies. Fleiss’s multi-rater kappa coefficient was used to assess the overall level of agreement between experts. The final consensus annotations were used as gold standard annotations evaluating the accuracy of caries diagnosis by the participants in the study, ensuring that both the presence/absence and specific caries diagnosis were clearly documented.

### Cognitive aids

**Figure 2.**
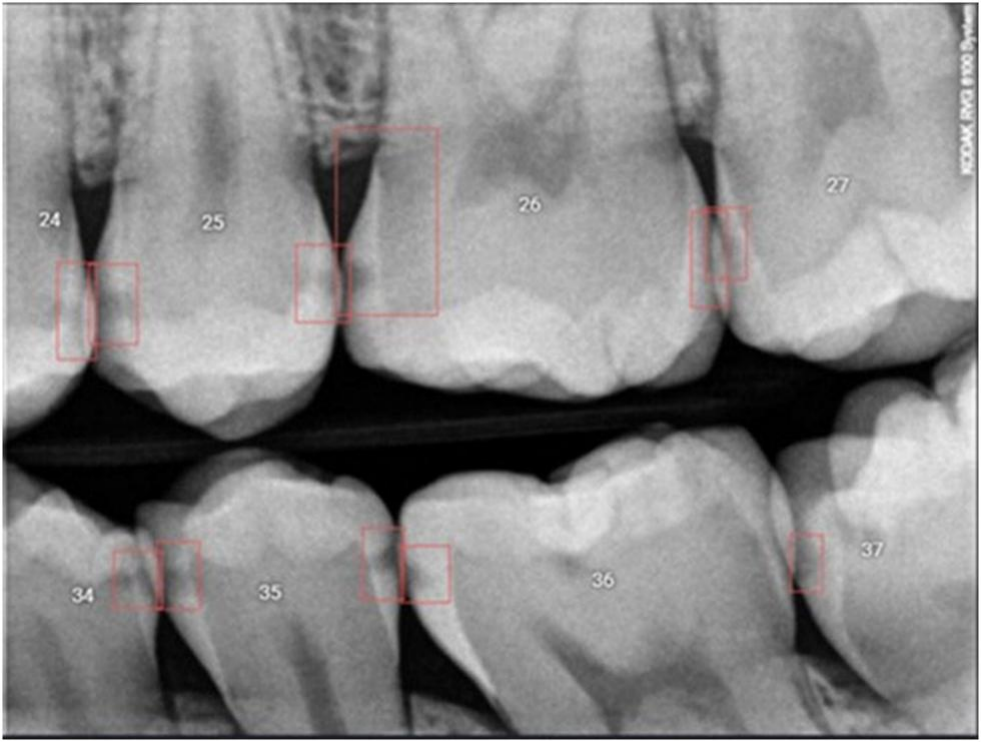
Bitewing radiograph with ML annotations identifying carious sites.

The research team developed a diagnostic checklist using international guidelines^40^ to assist the participants in systematically analysing the bitewing for caries identification. The checklist is included in Supplementary File S1. From the web search results for an automated caries detection program, companies that marketed ML algorithms for caries detection were contacted, and www.denti.ai (Denti.AI Technology Inc., Toronto, Canada) was chosen as they supported this study. Trial access to the platform was granted in July 2023. This FDA-approved ML algorithm identified areas of suspected caries on bitewing radiographs,^41^ but did not provide an ICDAS II grading to the participants in the ML group. Instead, it functioned as a caries detection tool (caries present/absent), assisting participants by highlighting regions of interest on the radiographs using a bounding box (Figure 2). The detection model was previously validated by internal testing and clinical studies, and according to www.denti.ai, it demonstrates high sensitivity and specificity for caries detection. However, it is important to note that the model’s performance may vary depending on image quality, variations in dental anatomy and operator interpretation.

The denti.ai algorithm is based on deep convolutional neural networks (CNNs) and integrates multiple specialized AI modules. According to García-Cañas et al^42^ and Tuzoff et al,^43^ the denti.ai system employs a Faster Region based CNN (Faster R-CNN) module for dental structure detection, a Visual Geometry Group (VGG)-16-based CNN architecture for tooth classification and numbering and a grayscale-based coding system for pathology detection. These models were trained on large, annotated dental radiograph datasets, enabling high sensitivity and specificity for interproximal caries detection.

While the detailed training dataset and internal architecture remain proprietary, independent studies have validated denti.ai’s diagnostic performance. One study tested the software on 300 bitewing radiographs and found diagnostic accuracies ranging from 70.8%-86.1% with a specificity up to 98.5% and sensitivity up to 87%, depending on the threshold model applied.^42^

### Study procedure

The study included 10 bitewing radiographs, selected based on quality criteria and randomized (not presented in order of difficulty) to eliminate the cognitive impairment effect due to image order. The bitewing images were displayed on iPad devices using the Qualtrics platform, and all participants completed their analyses in the same environment with controlled ambient lighting. Image enhancement tools were not used in this study, ensuring that all participants assessed the radiographs under standardized viewing conditions without additional enhancements.

Participants were randomly assigned to 3 groups by a participant randomizer created using a Python script (Supplementary File S3), ensuring that each participant had an equal chance of being assigned to any group. The Python code was designed to randomly allocate participants (using the “allocate_participants” function) into 3 groups, ensuring a 1:1:1 ratio. Each participant was given a participant reference number, and all collected data were anonymized. Group 1 was the control group, and the bitewings for caries were analysed without the help of a cognitive aid. Group 2 interpreted the same set of bitewing radiographs with the assistance of a caries detection machine learning algorithm. Group 3 received the same images for caries interpretation and was assisted by a checklist.

**Figure 3.**
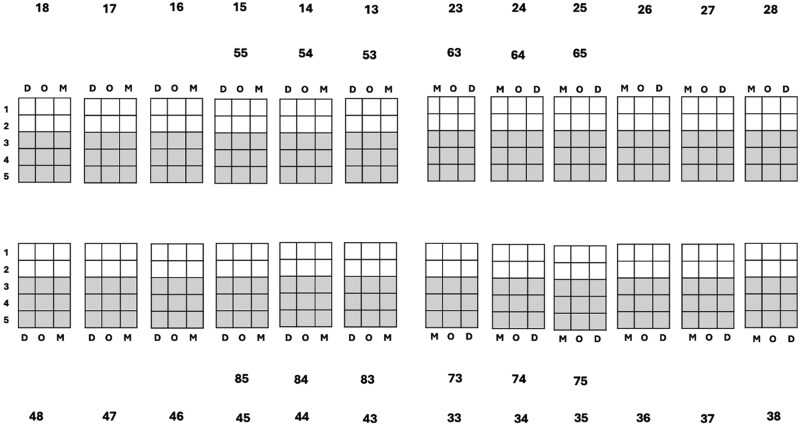
The diagnostic odontogram used to chart radiographic caries depth.

For each bitewing radiograph, participants noted whether the caries were present or absent. The participants further classified caries using the 5-point ICDAS II grading, which was recorded on a paper-based diagnostic odontogram, shown in Figure 3. Following caries diagnosis for each image, participants were asked to rate their confidence in the caries diagnosis using a 5-point Likert scale (1 = not at confident to 5 = extremely confident). This confidence score was collected for each bitewing.

### Questionnaire

At the end of the caries charting task, all participants completed an anonymized post-task questionnaire to record their feedback on their experience. The questions in the questionnaire varied depending on the group to which the participants were assigned. The questionnaire data were collected using Qualtrics (University of Sydney) (www.qualtrics.com), an online survey platform suitable for collecting and storing confidential and highly confidential data. All other data were stored in the University’s OneDrive password-protected account.

The questionnaire was used to identify (1) the participant’s views on how helpful the cognitive aids were in assisting with caries diagnosis and (2) the participants’ attitude towards cognitive aids—whether they would consider using them in future clinical practice.

**Figure 4.**
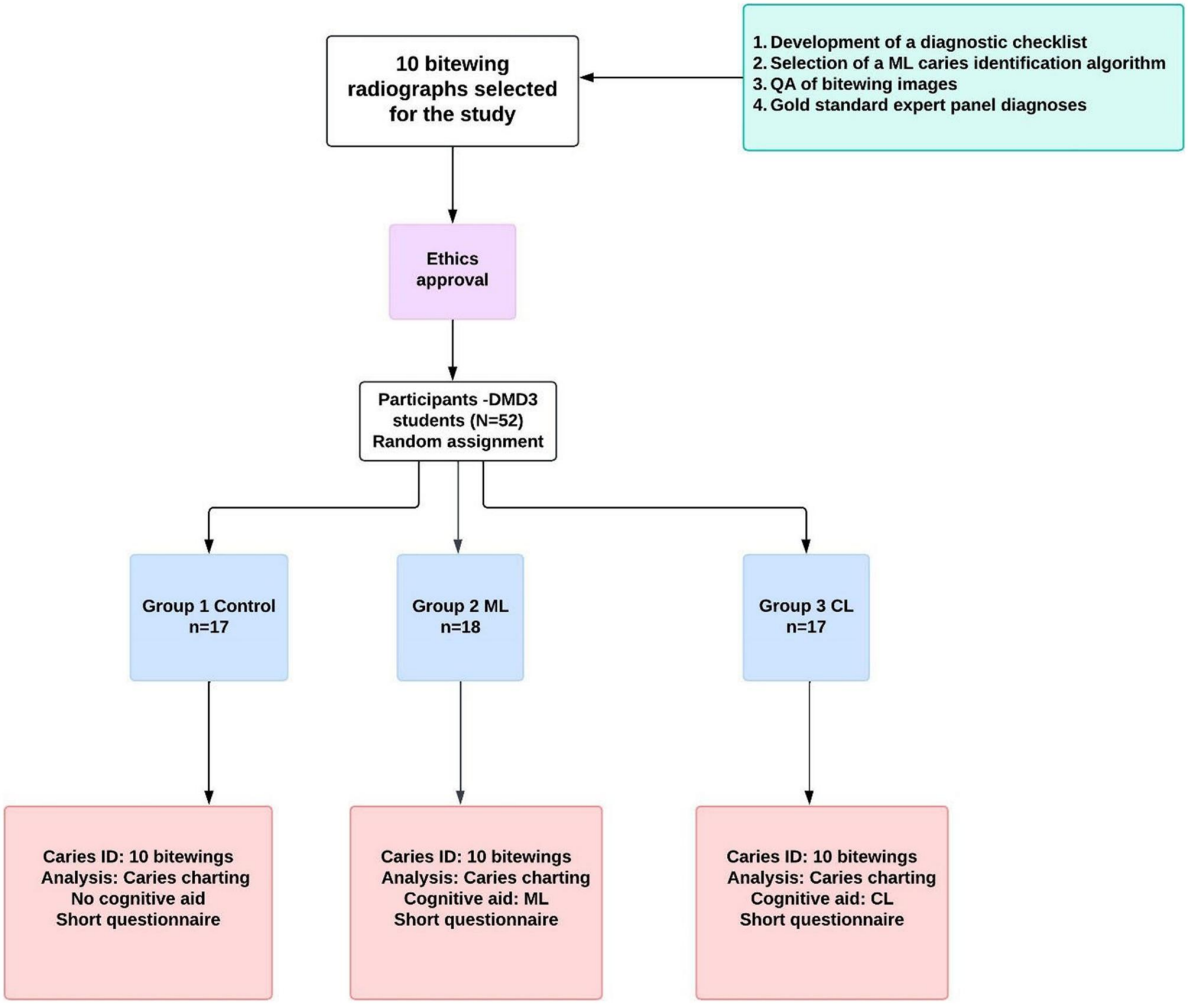
Flowchart illustrating the study procedures. ML = machine learning; QA = quality assessment; DMD = Doctor of Dental Medicine; CL = checklist.

The questionnaires are included in Supplementary File S2. The steps in the study are shown in Figure 4.

### Data analysis

Statistical analysis was performed using IBM SPSS statistics (IBM Corp. Released 2023. IBM SPSS Statistics for Windows, Version 26.0. Armonk, NY: IBM Corp).

Descriptive statistics were used to summarize participant demographic data, including frequency, percentages and measures of central tendency and variability. The interquartile range method was used to remove any outliers in descriptive data records before analysis.^44^

The diagnostic performance for caries detection (caries present/caries absent) was evaluated using sensitivity (or recall; measures the ability to identify caries correctly), specificity (measures the ability to correctly identify the absence of caries), and diagnostic odds ratio (DOR; summarizes the overall diagnostic performance) (Table S1). The inter-rater agreement between participants was compared for caries detection (caries present/absent) using Fleiss’s kappa. The ordinal ICDAS II scores (caries charting) were analysed using Cohen’s weighted kappa to assess agreement between the participant’s ICDAS II and the gold standard. Agreement between the 3 groups (control, CL, and ML) was also compared using weighted kappa. The weighted kappa method accounts for ordinal data by assigning smaller penalties for minor disagreements (eg ICDAS II scores 1 vs 2) and larger penalties for major disagreements (eg ICDAS II scores 2 vs 5), ensuring that more severe misclassifications contribute more heavily to the overall measure of disagreement. The confidence ratings (measured on a 5-point Likert scale) were compared between groups using the Kruskal-Wallis test. *Post hoc* pairwise comparisons were performed using Dunn’s test with a Bonferroni correction to identify significant differences.

The chi-square test of independence was used to compare categorical data between the groups with Fisher’s exact test when cell counts were low. For continuous variables such as age and the time taken for radiographic diagnosis, which were not normally distributed, the non-parametric independent-samples Kruskal-Wallis test was used to compare those between the study groups.

The post-task questionnaire data were analysed using descriptive statistics.

## Results

### Participant characteristics

Fifty-two students enrolled in academic year 3 of the DMD programme (27 females, 24 males; mean age 24.7 ± 4.3 years) at Sydney Dental School participated in this study. Age and duration taken for image analysis were the same for the control, ML, and CL groups (*P* > .05) (Table S2).

### Development of gold standard

From the 10 bitewing images, 223 tooth surfaces were assessed (150 interproximal surfaces and 73 occlusal surfaces). Of these surfaces, 56 had caries, and 167 surfaces were caries free. The images and details about the number and severity of caries (based on ICDAS II) are available in Supplementary File S4. Expert consensus formed the gold standard, with substantial agreement (Fleiss’s κ = 0.707, 95% CI: 0.682-0.732; *P* < .001) (Based on Landis and Koch’s interpretation scale,^45^  *<0.00: poor agreement; 0.00-0.20: slight agreement; 0.21-0.40: fair agreement; 0.41-0.60: moderate agreement; 0.61-0.80: substantial agreement; 0.8-1.00: almost perfect agreement)*.

### Diagnostic performance of caries detection across study groups

For dichotomous (caries present/absent) caries grading, ML group achieved the highest sensitivity (79.1%), followed by the control group (67.9%) and the CL group (63.9%) (*P* < .001). In contrast, the CL group (90.9%) had the highest specificity, followed by the control group (87.6%) and the ML group (84.3%) (*P* < .001). The ML group had the highest DOR of 20.3, (*P* < .001), followed by the CL group with a DOR of 17.7, and the control group with the lowest DOR at 15.0 (*P* < .001) (Table 1).

**Table 1.** Diagnostic performance using dichotomous caries grading for ML, control and checklist groups.

	Study group					
Diagnostic test	Control (*n* = 17)		ML (*n* = 18)		CL (*n* = 17)	
	Estimate	95% CI	Estimate	95% CI	Estimate	95% CI
Sensitivity (%)	67.9	64.9-70.7	79.0	76.5-81.4	63.9	60.9-66.9
Specificity (%)	87.7	85.9-89.4	84.3	82.4-86.2	90.9	89.3-92.4
Diagnostic odds ratio	15.0	12.2-18.6	20.3	16.5-24.9	17.7	14.1-22.3

Abbreviations: CI = confidence interval; CL = checklist; ML = machine learning.

Overall, the ML group showed superior performance on caries detection across sensitivity and diagnostic odds ratio. The CL group had the highest specificity among the three groups, but had lower scores for other diagnostic metrics compared to the ML group. When the performance of CL and the control groups were compared, the CL group had higher specificity and DOR compared to the control group. However, the control group performed better in sensitivity.

### Inter-rater agreement for caries diagnosis

For the caries detection task, the ML group demonstrated the highest agreement among participants (Fleiss’s κ = 0.702, 95% CI: 0.692-0.713; *P* < .001), indicating substantial agreement. The checklist group followed with a moderate agreement (κ = 0.604, 95% CI: 0.593-0.615; *P* < .001), and the control group showed the lowest agreement (κ = 0.590, 95% CI: 0.578-0.601; *P* < .001), also indicating moderate agreement.

For the ICDAS II scoring task, the ML group demonstrated the highest agreement with the gold standard (Cohen’s κ = 0.520, *P* < .001), followed by the control group (κ = 0.476, *P* < .001), and the checklist group showed the lowest agreement (κ = 0.456, *P* < .001). (*<0.00 indicates poor agreement; 0.00-0.20: slight agreement; 0.21-0.40: fair agreement; 0.41-0.60: moderate agreement; 0.61-0.80: substantial agreement; 0.8-1.00: almost perfect agreement*).

### Confidence in caries diagnosis

**Figure 5.**
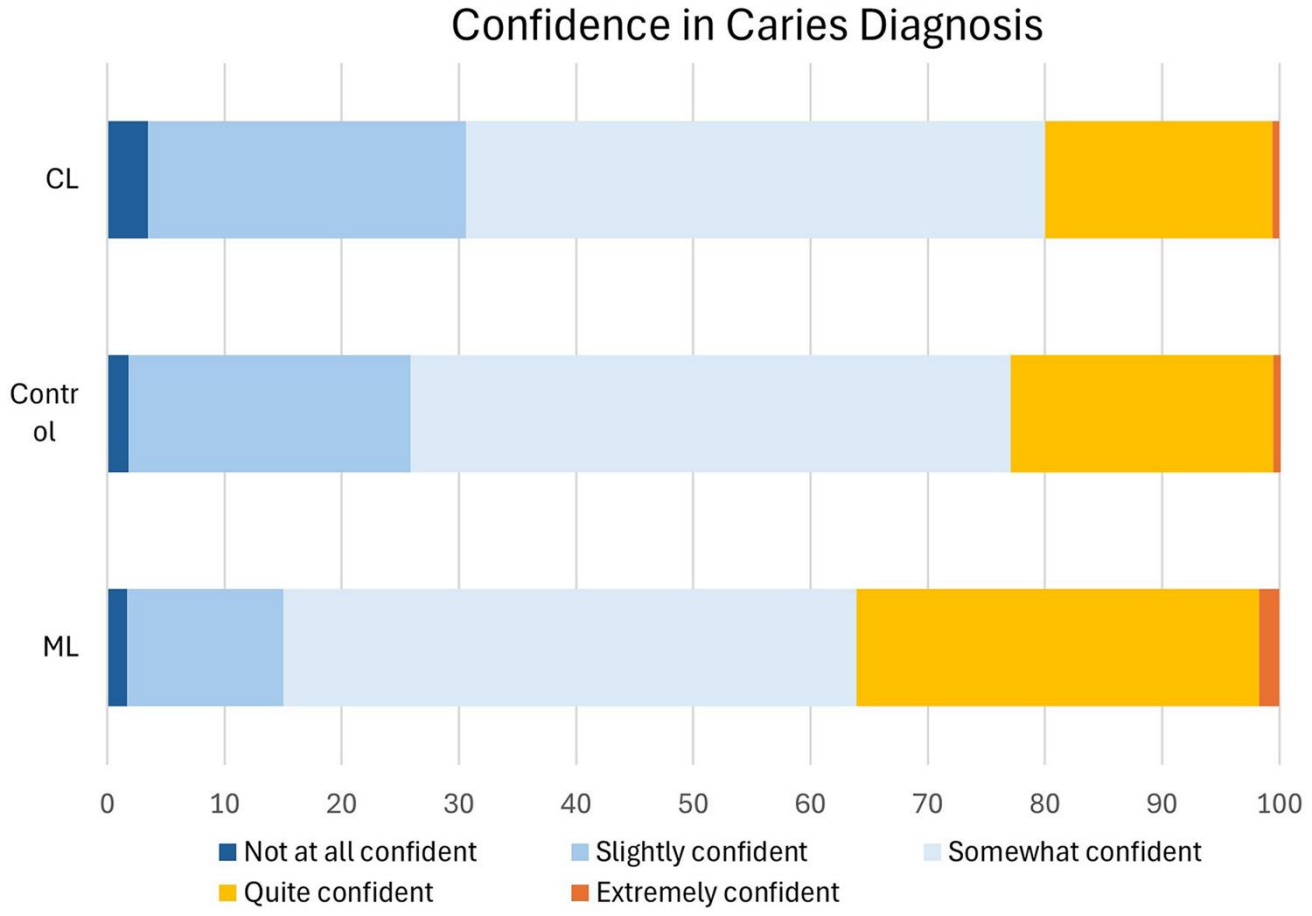
Confidence in caries diagnosis for the 3 groups.

A higher proportion of participants in the ML group reported greater confidence (quite confident/extremely confident) compared to the other 2 groups. Confidence levels varied across the different images for all groups, suggesting that some images were inherently more challenging or easier to interpret, regardless of the tool or method used (Figure 5).

To compare confidence levels between the 3 groups, a Kruskal-Wallis test was performed, revealing a statistically significant difference between the 3 groups (H (2) = 21.937, *P* < .001). *Post hoc* Dunn’s pairwise comparisons with Bonferroni correction showed the ML group had significantly higher confidence scores compared to the control (*P* < .001) and checklist groups (*P* < 0.001). However, there were no significant differences in confidence scores between the control and checklist groups (*P* = .918).

Descriptive statistics were calculated to compare confidence scores across the 3 groups (control, checklist, and ML). The ML group had the highest mean confidence score (M = 3.23, SD 0.77), followed by the control group (M = 2.96, SD 0.75) and the checklist group had the lowest mean confidence (M = 2.86, SD 0.78). Median confidence scores were equal across all groups (Mdn = 3, IQR 1), but the confidence interval of the ML group did not overlap with those of the checklist group, indicating meaningful differences in confidence levels.

A majority of the participants across the 3 groups reported being “somewhat confident”. However, the higher mean score in the ML group suggests that a higher proportion of participants reported higher confidence levels (quite confident or extremely confident).

### Post-task questionnaire regarding the use of cognitive aids in caries diagnosis

Participants’ views about their willingness to use the cognitive aids and their impact on diagnosis were assessed. Participants could choose more than one response to several questions.

In the ML group (*n* = 18), 72.2% reported that they used the ML prompts to validate their diagnosis and 55.6% as a second opinion. A majority (88.2%) in the control group (*n* = 17) indicated they would consider using ML in future clinical practice. Similarly, in the checklist group (*n* = 17), 58.8% used the checklist to feel more confident in their diagnosis, though perceptions of usefulness of checklists were divided with 41.2% finding it useful and 41.2% finding it not useful.

Regarding the agreement in diagnosis with the ML prompt, 50% of the participants found that their caries diagnosis aligned with the ML prompt, while 27.8% indicated that they changed their diagnosis based on the ML prompt. Data were missing for 22.2% of the participants.

A significant majority of the participants (88.2%) did not provide reasons for using the checklist. When asked if the checklist was helpful, responses were evenly split, with 41.2% of the participants finding it helpful and 41.2% of the participants stating that it was not helpful, with data missing for 17.6% of participants.

Detailed response distributions are available in Table S3.

## Discussion

This study compared the diagnostic accuracy and inter-rater reliability among 3 groups of dental students- control, machine learning (ML), and checklist (CL) groups at 2 thresholds: caries identification of bitewing radiographs (detection) and the more detailed ICDAS II grading of caries severity. The findings suggest that the ML assisted diagnosis improved overall diagnostic accuracy and confidence, highlighting the potential benefits of AI based decision support in radiographic analysis.

One of the key findings was that the ML may have enhanced diagnostic performance, possibly by reducing cognitive load. Radiographic interpretation can be a cognitively demanding task and requires clinicians to analyse complex visual data while maintaining diagnostic consistency. The ML tool likely functioned as a cognitive scaffold, allowing participants to focus their attention on high-risk areas, verify ambiguous cases and make more confident decisions. This is supported by the higher confidence levels reported by ML users, suggesting that ML tools can enhance decision making by providing real-time validation of clinical judgements. Future studies should employ objective measures to further investigate the specific cognitive processes involved, such as visual attention, decision latency or mental effort to better understand the mechanisms through which ML supports radiographic interpretation.

While the ML group demonstrated lower specificity compared to the checklist group, it achieved higher sensitivity, diagnostic odds ratio (DOR) and confidence levels. The high DOR observed in the ML group reflected a strong diagnostic performance, with superior discrimination between carious and non-carious surfaces, reinforcing its value as a diagnostic aid in radiographic interpretation of dental caries. The trade-off between sensitivity and specificity is an important consideration in diagnostic performance. Higher sensitivity means that ML is better at detecting caries, reducing the likelihood of missed lesions that could progress without intervention. However, this can come at the cost of increased false positives, where sound surfaces may sometimes be misclassified as carious. In populations with moderate to high prevalence of caries, such as the Australian adult population, where 1 in 3 adults have at least one untreated carious tooth surface,^13^ specificity becomes particularly important to avoid overdiagnosis and unnecessary treatment. Overdiagnosis can lead to unwarranted treatment, increased patient costs, and loss of sound tooth surface. Additionally, unnecessary treatment places an extra burden on already strained public healthcare resources, particularly in rural and remote areas, where access to dental care is limited. Importantly, reducing cognitive load and optimizing specificity are not mutually exclusive goals. A clinician centred approach that integrates ML as a support tool rather than as a replacement for human expertise ensures a balanced and effective diagnostic process. Future research should focus on ensuring that ML models effectively support accurate, efficient and resource conscious clinical decision making. In contrast, the checklist group exhibited higher specificity, meaning it was more conservative in diagnosing caries, potentially reducing unnecessary treatment, but this came at the cost of lower sensitivity, potentially leading to missed diagnosis.

Interestingly, the control group had higher sensitivity than the checklist group, indicating that participants relying solely on their clinical judgements were more likely to classify surfaces as carious rather than missing potential lesions. In contrast, the checklist group may have faced challenges due to the initial learning curve associated with checklists.^46^ Given their relative inexperience, our participants may not have fully adapted to incorporating checklists into their diagnosis in the study. Additionally, checklists can sometimes lead to a mechanical approach to diagnosis, or the clinician may decide to skim or not entirely follow the checklists.^47^ The unfamiliarity of the checklist may have contributed to the poorer performance in the checklist group in our study. As a result, checklists may not have been as effective as expected in reducing cognitive load and improving diagnostic accuracy. However, studies have shown that the usefulness of checklists may be improved by using digital checklists and relatively uncomplicated designs based on minimalist principles.^48^

While our results demonstrate statistically significant differences in diagnostic accuracy between groups, the overlapping confidence intervals suggest caution in interpreting these differences as definitive. For instance, the confidence intervals for sensitivity in the ML group do not overlap, clearly demonstrating meaningful improvement. However, the intervals for specificity for the 3 groups partially overlap, indicating that the practical differences in avoiding false positives may be less distinct. This suggests that while ML-assisted diagnosis enhances sensitivity, further refinement of these algorithms is necessary to balance sensitivity and specificity.

Confidence in diagnosis is crucial in clinical settings where diagnostic uncertainty may increase cognitive load and affect clinical decision-making.^49^ By automating the analysis and interpretation of dental radiographs, ML algorithms can provide insights and decision support by highlighting areas of dental caries.^50^ This can help clinicians focus their attention effectively and reduce the likelihood of missed diagnoses. As a result, ML algorithms can free up cognitive resources that clinicians can allocate to other critical aspects of patient care. This suggests that ML algorithms can act as a valuable second opinion, validating the clinician’s diagnosis and reassuring them. This increased confidence is particularly beneficial in clinical settings where diagnostic uncertainty can impact decision-making and patient outcomes. While studies in medicine discuss the impact of ML on diagnostic performance,^51^^,^^52^ the influence of ML algorithms on clinician confidence in diagnosis has not been studied, particularly in dentistry. The checklist group, despite using a structured aid, did not experience the same confidence boost, possibly due to the additional cognitive steps required to use checklists effectively.

The questionnaire data provided valuable insights into the participants’ experiences and attitudes towards using cognitive aids in dental caries diagnosis. The ML group’s responses highlight the potential of ML algorithms to enhance diagnostic confidence and accuracy, especially as a second-opinion tool. Overall, the questionnaire responses indicate the need for user-friendly cognitive aids that can cater to novice and experienced clinicians.

This study was not without limitations. The bitewing images included in this study had minimal technical issues (film faults), such as interproximal overlap. This may not reflect the technical variability commonly encountered in clinical practice. The selection of bitewings in this study may have improved the diagnostic performance for all groups, including the ML group. Future studies should evaluate ML performance in realistic settings by incorporating images with film faults commonly encountered in general dentistry. While the image dataset consisted of a range of caries severities classified according to the ICDAS radiographic criteria, we acknowledge that early caries (ICDAS codes 1 and 2) was under-represented in the sample. This could have influenced the diagnostic performance, as early lesions are inherently more difficult to identify radiographically. Future studies will consider oversampling early enamel lesions to more accurately assess the diagnostic utility of cognitive aids in detecting early caries.

This study considered the impact of cognitive aids on the diagnosis of caries, and the participants did not have to provide treatment to the patients based on the diagnosis. In that sense, this study could be considered “low stakes”, and the participants’ diagnostic decisions did not impact patients. Additionally, the study participants were third-year dental students with limited clinical experience. Less experienced participants may be more open to ML prompts due to their need for greater support, whereas experienced clinicians are likely to rely on their own expertise and judgement. While the results are promising, the relatively small sample size and selective nature of the study participants, consisting of third-year dental students from a single dental school may limit the generalizability of the study findings. While previous studies guided the sample size calculation,^53^ future studies should refine these calculations through preliminary pilot studies or meta-analyses to ensure precise power calculations. The study utilized a specific ML algorithm for caries detection. The performance and user acceptance of different ML tools may vary, and we acknowledge that sensitivity and specificity can differ significantly across algorithms due to differences in architecture, training datasets, and labelling standards.^54^ These variations can substantially influence diagnostic rates, especially in identifying early carious lesions or borderline cases. While comparative evaluations of multiple ML models are valuable, the aim of this study was not to benchmark or validate the influence of a single ML based algorithm on dental student decision making. In addition, participants’ self-reported confidence levels, introducing a subjective element to the data. Individual biases can influence self-assessment and may not accurately reflect true diagnostic confidence or competence.

Future studies will include larger sample sizes with diverse participants, clinicians with varying clinical experience and multicentre studies. Further research is also required to compare the impact of ML algorithms on the diagnostic accuracy of a range of dental conditions on various types of dental imaging, such as panoramic and Cone Beam Computed Tomography (CBCT) images. It would also be worthwhile to conduct such studies in clinical settings where the treatments are provided to patients based on the input and support provided by cognitive aids. Furthermore, it may be valuable to incorporate clinical examination and operative findings, such as lesion depth on caries removal, into the reference standard to complement radiographic assessments and improve diagnostic certainty. Testing the effectiveness of cognitive aids under conditions of increased cognitive load, such as complex cases and time pressure would also offer insights into their real world utility.

## Conclusion

Cognitive aids, particularly machine learning algorithms, can enhance diagnostic accuracy and clinician confidence while diagnosing dental caries from bitewing radiographs. Integrating them into dental education and clinical practice can shape the diagnostic capabilities of current and future practitioners, leading to consistent and reliable diagnoses and ultimately enhancing patient care. Investing in comprehensive training ensures dental students and clinicians can effectively utilize AI tools to improve diagnostic accuracy by reducing cognitive load.

## Supplementary Material

twaf053_Supplementary_Data
