# Supplementary material for: Machine learning algorithms enhance the accuracy of radiographic diagnosis of dental caries: a comparative study
Source: Dentomaxillofac Radiol. 2025 Jul 10;54(8):632–41. doi: 10.1093/dmfr/twaf053 (PMC12653770; doi:10.1093/dmfr/twaf053)
Supplement: twaf053_Supplementary_Data [file twaf053_supplementary_data.zip › Supplementary File 2.docx]

Post task questionnaire:

**Post-task questionnaire/ Dental students**

**Survey Flow**

**Block: Default Question Block (6 Questions)**

**Branch: New Branch**

**If**

**If Which arm of the study did you participate in? Group 2/ML Is Selected**

**Block: ML (9 Questions)**

**EndSurvey:**

**Branch: New Branch**

**If**

**If Which arm of the study did you participate in? Group 1/ NA Is Selected**

**Block: NA (6 Questions)**

**EndSurvey:**

**Branch: New Branch**

**If**

**If Which arm of the study did you participate in? Group 3/CL Is Selected**

**Block: CL (6 Questions)**

**EndSurvey:**

| Page Break |  |
| --- | --- |

**Start of Block: Default Question Block**

Q1 Please enter your participant ID

________________________________________________________________

Q21 Please state your age

________________________________________________________________

Q22 Gender

- Male (4)
- Female (5)
- Non-binary / third gender (6)
- Prefer not to say (7)

Q32 Please state your year of study

- DMD 2 (1)
- DMD 3 (2)
- DMD 4 (3)

Q23 What were your undergraduate qualifications?

- Oral health/dentistry (3)
- Non oral health (4)

Q3 Which arm of the study did you participate in?

- Group 1/ NA (1)
- Group 2/ML (2)
- Group 3/CL (3)

**End of Block: Default Question Block**

**Start of Block: ML**

Q8 Did you refer to the prompts generated by the machine learning (ML) algorithm to reach your caries diagnosis ?

- Yes (1)
- No (3)

*Display This Question:*

*If Did you refer to the prompts generated by the machine learning (ML) algorithm to reach your carie... = Yes*

Q24 When did you use the prompts provided by the ML algorithm in your diagnostic process? 

- Before looking at the radiograph, I checked the prompts to reach a diagnosis (1)
- After looking at the radiograph, I checked the prompts to assist me with the diagnosis (2)
- After looking at the radiograph, I checked the prompts to confirm my diagnosis (3)
- After looking at the radiograph, I used the prompts to reach a diagnosis (4)
- After recording my diagnosis, I checked the prompts, but it didn't affect my decisions (5)

*Display This Question:*

*If When did you use the prompts provided by the ML algorithm in your diagnostic process?  = Before looking at the radiograph, I checked the prompts to reach a diagnosis*

*Or When did you use the prompts provided by the ML algorithm in your diagnostic process?  = After looking at the radiograph, I checked the prompts to assist me with the diagnosis*

*Or When did you use the prompts provided by the ML algorithm in your diagnostic process?  = After looking at the radiograph, I checked the prompts to confirm my diagnosis*

*Or When did you use the prompts provided by the ML algorithm in your diagnostic process?  = After looking at the radiograph, I used the prompts to reach a diagnosis*

*Or When did you use the prompts provided by the ML algorithm in your diagnostic process?  = After recording my diagnosis, I checked the prompts, but it didn't affect my decisions*

Q9 Why did you refer to the prompts given by ML algorithm? (multiple options may apply)

- It saves time (1)
- I was not confident to interpret the images (2)
- I trust the prompts made by the ML (3)
- To validate my diagnosis (4)
- As a second opinion (5)
- Other, please specify (6) __________________________________________________

*Display This Question:*

*If Did you refer to the prompts generated by the machine learning (ML) algorithm to reach your carie... = No*

Q26 Why did you not refer to the prompts given by ML algorithm? (multiple options may apply)

- It wastes time (1)
- I was confident to interpret the images (2)
- I don't trust the prompts made by the ML (3)
- I did not like the user interface (4)
- I don't think the prompts by ML algorithm are accurate (5)
- Other, please specify (6) __________________________________________________

*Display This Question:*

*If Why did you refer to the prompts given by ML algorithm? (multiple options may apply) = It saves time*

*Or Why did you refer to the prompts given by ML algorithm? (multiple options may apply) = I was not confident to interpret the images*

*Or Why did you refer to the prompts given by ML algorithm? (multiple options may apply) = I trust the prompts made by the ML*

*Or Why did you refer to the prompts given by ML algorithm? (multiple options may apply) = To validate my diagnosis*

*Or Why did you refer to the prompts given by ML algorithm? (multiple options may apply) = As a second opinion*

*Or Why did you refer to the prompts given by ML algorithm? (multiple options may apply) = Other, please specify*

Q12 Was your caries diagnosis in agreement with the prompts by the ML algorithm?

- Yes (1)
- No (2)

*Display This Question:*

*If Was your caries diagnosis in agreement with the prompts by the ML algorithm? = No*

Q29 Did you change your diagnosis after seeing the ML prompt?

- Yes (1)
- No (2)

*Display This Question:*

*If Did you change your diagnosis after seeing the ML prompt? = Yes*

Q27 Why did  you choose the ML prompt over your diagnosis?

________________________________________________________________

*Display This Question:*

*If Did you change your diagnosis after seeing the ML prompt? = No*

Q28 Why did you choose your diagnosis over the ML prompt?

________________________________________________________________

*Display This Question:*

*If Was your caries diagnosis in agreement with the prompts by the ML algorithm? = Yes*

*Or Or Why did you choose the ML prompt over your diagnosis? Text Response Is Displayed*

*Or Or Why did you choose your diagnosis over the ML prompt? Text Response Is Displayed*

Q13 Did the ML prompts affect your confidence in your diagnosis?

- No (2)
- Sometimes (3)

**End of Block: ML**

**Start of Block: NA**

Q15 Would you consider using a checklist to assist with caries identification?

- Yes (1)
- No (2)

*Display This Question:*

*If Would you consider using a checklist to assist with caries identification? = Yes*

Q16 If yes, please select your reason(s) from this list

- It saves time (1)
- It prevents lesions from being missed (2)
- Makes the image interpretation more systematic (3)
- Other (4) __________________________________________________

*Display This Question:*

*If Would you consider using a checklist to assist with caries identification? = No*

Q33 If no, please select your reason(s) from the list

- It is inefficient (1)
- I don't need a checklist to help me (2)
- Wastes time (3)
- Other (4) __________________________________________________

Q17 Would you consider using machine learning algorithm to assist you with caries identification?

- Yes (1)
- No (2)

*Display This Question:*

*If Would you consider using machine learning algorithm to assist you with caries identification? = Yes*

Q18 If yes, please select your reason(s) from the list

- It saves time (1)
- I am not confident to interpret the images (2)
- I trust the prompts made by the ML algorithm (3)
- To validate my diagnosis (4)
- As a second opinion (5)
- Other (6) __________________________________________________

*Display This Question:*

*If Would you consider using machine learning algorithm to assist you with caries identification? = No*

Q34 If no, please select your reason(s) from the list

- It wastes time (1)
- I am confident to interpret the images on my own (2)
- I don't trust the prompts given by the ML (3)
- I don't the user interface (4)
- I don't think the prompts by ML algorithm are accurate (5)
- Other (6) __________________________________________________

**End of Block: NA**

**Start of Block: CL**

Q2 Did you use the checklist to reach your caries diagnosis?

- Yes (1)
- No (3)

*Display This Question:*

*If Did you use the checklist to reach your caries diagnosis? = Yes*

Q4 When did you use the checklist?

- Before looking at the radiograph (1)
- After looking at the radiograph, I used the checklist to assist me in diagnosis (2)
- After looking at the radiograph, I used the checklist to confirm my diagnosis (4)
- After recording my diagnosis, I used the checklist but it didn't affect my diagnosis (5)

*Display This Question:*

*If When did you use the checklist? = Before looking at the radiograph*

*Or When did you use the checklist? = After looking at the radiograph, I used the checklist to assist me in diagnosis*

*Or When did you use the checklist? = After looking at the radiograph, I used the checklist to confirm my diagnosis*

*Or When did you use the checklist? = After recording my diagnosis, I used the checklist but it didn't affect my diagnosis*

Q6 Why did you use the checklist?

- It saves time (1)
- It makes me more confident my diagnosis (2)
- It make the diagnosis more accurate (3)
- Other (4) __________________________________________________

*Display This Question:*

*If Why did you use the checklist? = It saves time*

*Or Why did you use the checklist? = It makes me more confident my diagnosis*

*Or Why did you use the checklist? = It make the diagnosis more accurate*

*Or Why did you use the checklist? = Other*

Q5 Did the checklist help you with the diagnosis?

- Yes (1)
- No (2)

*Display This Question:*

*If Did you use the checklist to reach your caries diagnosis? = No*

Q31 Why did you not use the checklist?

- It wastes time (1)
- I am confident about my diagnosis (2)
- It does not increase the accuracy of diagnosis (3)
- Other (4) __________________________________________________

*Display This Question:*

*If Did the checklist help you with the diagnosis? = Yes*

*Or Why did you not use the checklist? = It wastes time*

*Or Why did you not use the checklist? = I am confident about my diagnosis*

*Or Why did you not use the checklist? = It does not increase the accuracy of diagnosis*

*Or Why did you not use the checklist? = Other*

Q30 Did the checklist affect your confidence to diagnosing caries?

- Yes (1)
- No (2)

**End of Block: CL**
