# Supplementary material for: Machine learning algorithms enhance the accuracy of radiographic diagnosis of dental caries: a comparative study
Source: Dentomaxillofac Radiol. 2025 Jul 10;54(8):632–41. doi: 10.1093/dmfr/twaf053 (PMC12653770; doi:10.1093/dmfr/twaf053)
Supplement: twaf053_Supplementary_Data [file twaf053_supplementary_data.zip › Supplementary File 3.docx]

Python Code

>>> import random

>>> participants = ["Participant1", "Participant2", "Participant3", "Participant4", "Participant5", "Participant6", "Participant7", "Participant8", "Participant9", "Participant10", "Participant11" ]

>>> random.shuffle(participants)

... participants_per_group = len(participants) // 3

... group1 = participants[:participants_per_group]

... group2 = participants[participants_per_group:2 * participants_per_group]

... group3 = participants[2 * participants_per_group:]

... print("Group 1:", group1)

... print("Group 2:", group2)

... print("Group 3:", group3)
