# Supplementary material for: Machine learning algorithms enhance the accuracy of radiographic diagnosis of dental caries: a comparative study
Source: Dentomaxillofac Radiol. 2025 Jul 10;54(8):632–41. doi: 10.1093/dmfr/twaf053 (PMC12653770; doi:10.1093/dmfr/twaf053)

**Gold standard:**

The image set consisted of 10 bitewings with 150 interproximal surfaces and 73 occlusal visible. Of these, 56 surfaces had caries. Based on the gold standard diagnoses by experts the carious areas were classified as shown in the table below:

| Caries classification criteria | Number of surfaces | Total surfaces |
| --- | --- | --- |
| Code 0- no caries | 167 | 167 |
| Code 1- less than half of enamel | M-6; D-2; O-0 | 8 |
| Code 2-more than half of enamel | M-11; D-6; O-0 | 17 |
| Code 3- just into dentine | M-5; D-7; O-2 | 14 |
| Code 4-outer one-third of dentine | M-2; D-5; O-4 | 11 |
| Code 5- inner two-third of dentine | M-1; D-3; O-2 | 6 |

Image wise caries on 10 bitewings

| Image | Proximal surfaces visible | Occlusal surfaces visible | Caries | Caries free |
| --- | --- | --- | --- | --- |
| 1 | 18 | 8 | 6 | 20 |
| 2 | 16 | 8 | 4 | 20 |
| 3 | 12 | 6 | 1 | 17 |
| 4 | 12 | 7 | 1 (occlusal) | 18 |
| 5 | 18 | 8 | 4+1 occlusal | 21 |
| 6 | 20 | 8 | 8 | 20 |
| 7 | 12 | 7 | 10 +2 occlusal | 7 |
| 8 | 16 | 8 | 6 | 18 |
| 9 | 12 | 6 | 6 +3 occlusal | 9 |
| 10 | 14 | 7 | 3+1 occlusal | 17 |
| Total | 150 | 73 | 56 | 167 |

Caries identification- gold standard consensus

| Image number | Image type | Caries present |
| --- | --- | --- |
| 1 | Right bitewing showing 18-13 and 44-47 | 18-Mcode 2; 17 D code 2; 16 D code 4; 15 M code 3; 46 Mcode 2; 47 M code 1 |
| 2 | Left bitewing showing 23-27 and 34-37 | 26 D code 5; 27 M code 2; 35 D code 5; 37 M code 4 |
| 3 | Right bitewing showing 17-14 and 44-47 | 16 M code 1 |
| 4 | Left bitewing showing 24-27 and 34-37 | 27 occlusal code 3 |
| 5 | Right bitewing showing 17-13 and 43-47 | 16 M code 5; 14 D code 5; 14 O code 5; 45 D code 2; 46 M code 1 |
| 6 | Left bitewing showing 23-27 and 34-37 | 24 D code 2; 25 M code 1; 25 D code 2; 26 M code 2; 37 M code 1; 36 M code 2; 36 D code 1; 35 D code 1 |
| 7 | Left bitewing showing 24-27 and 34-37 | 24 D code 4; 25 D code 3; 25 M code 3; 26 M code 2; 27 M code 2; 37 M code 2; 37 O code 4; 36 M code2; 36 O code 4; 35 M code 3; D code 3; 34 D code 4 |
| 8 | Left bitewing showing 24-27 and 34-37 | 24 D code 2; 25 D code 4; 26 M code 2; 37 M code 3; 36 D code 3; 35 D code 3 |
| 9 | Left bitewing showing 24-27 and 36-38 | 24 D code 3; 25 D code3; 25 M code 3; 26 M code2; 27 O code 4; 37 O code 3; 37 M code 4; 36 O code 5; 36 D code 4 |
| 10 | Right bitewing showing 17-14 and 44-47 | 16 M code1; 15 D code 2; 45 D code 3; 47 O code 4 |

Image 1:


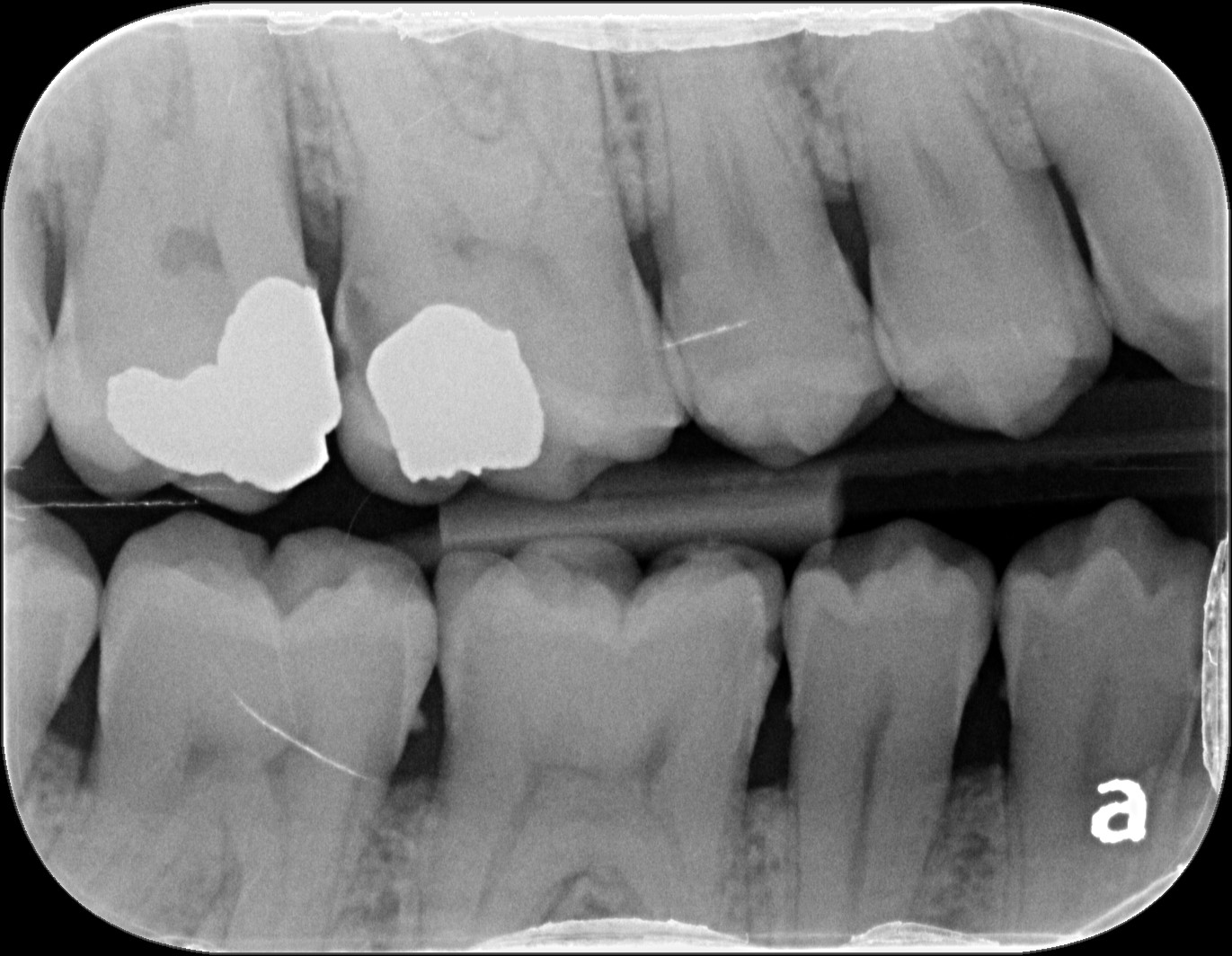


Image 2:


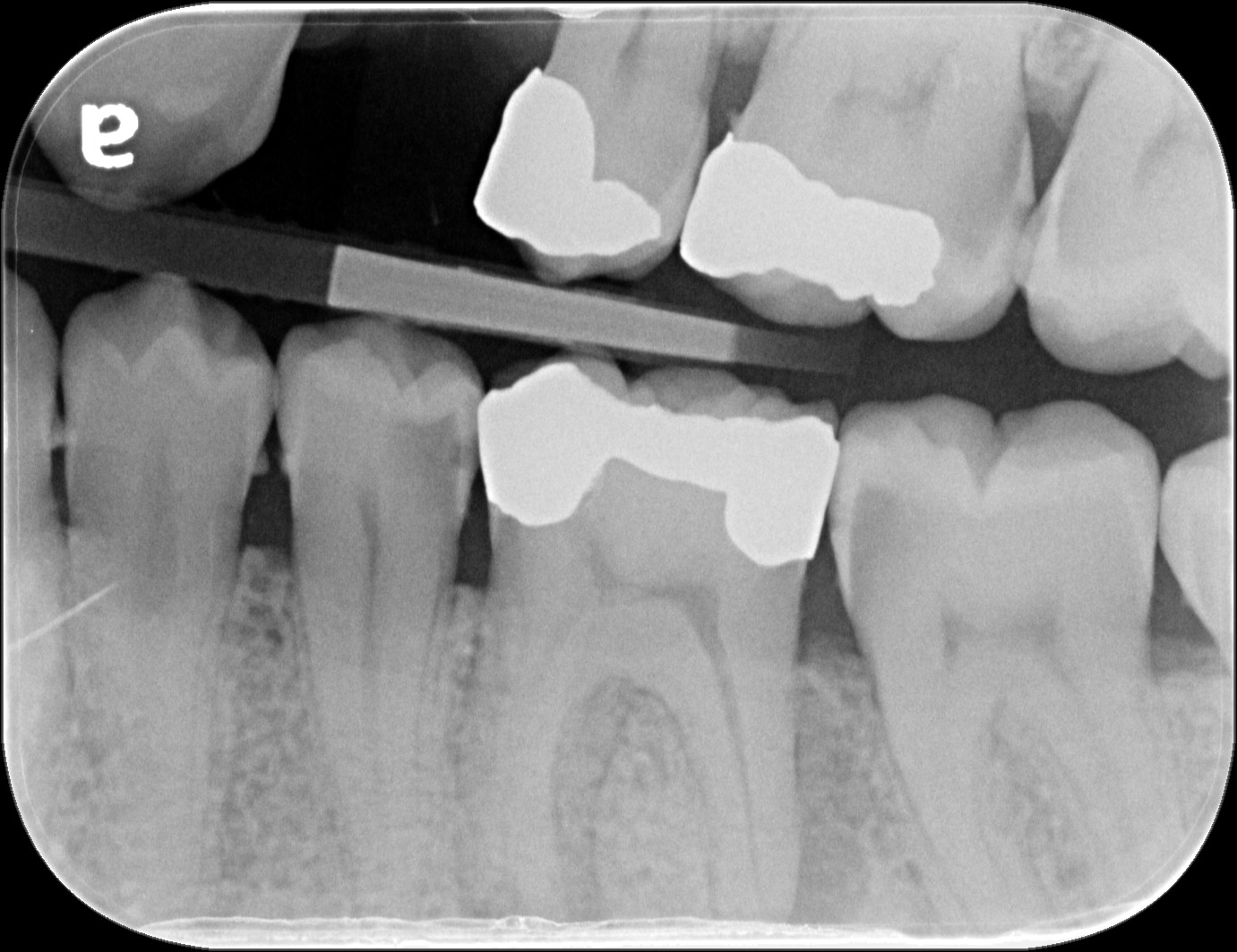


Image 3:


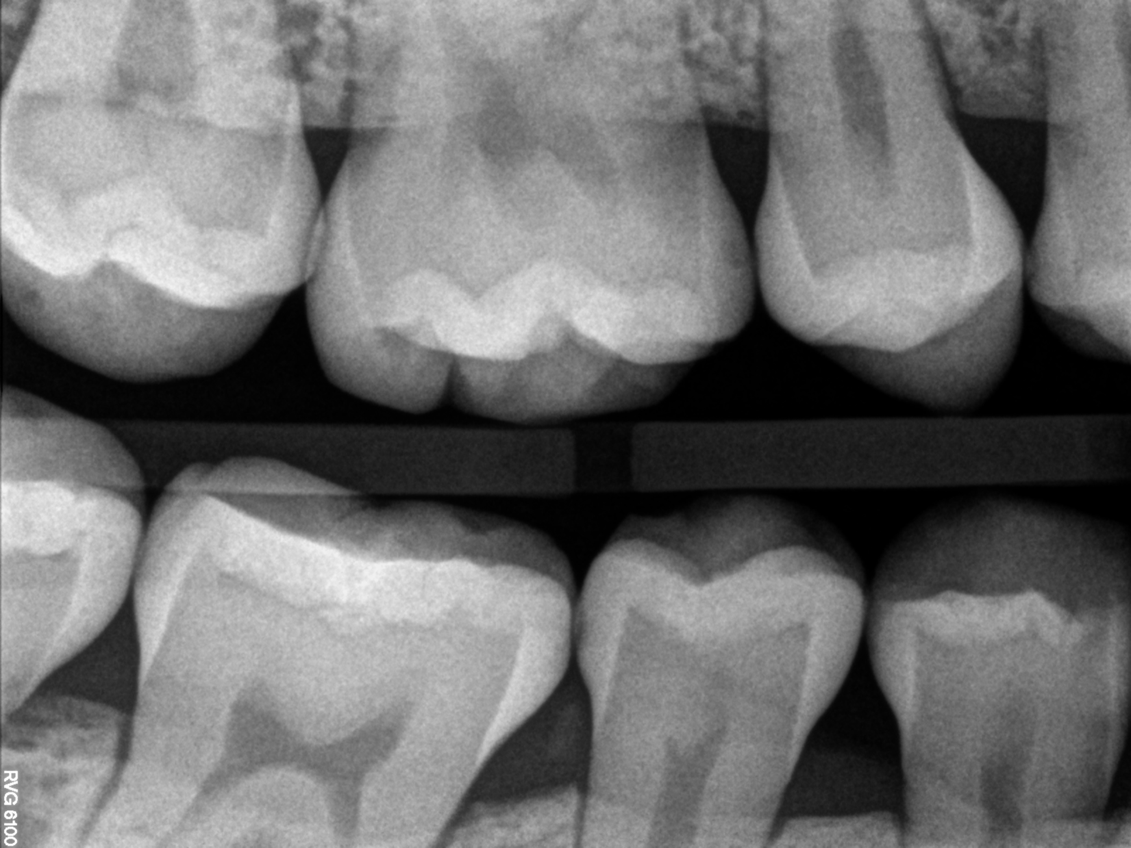


Image 4:


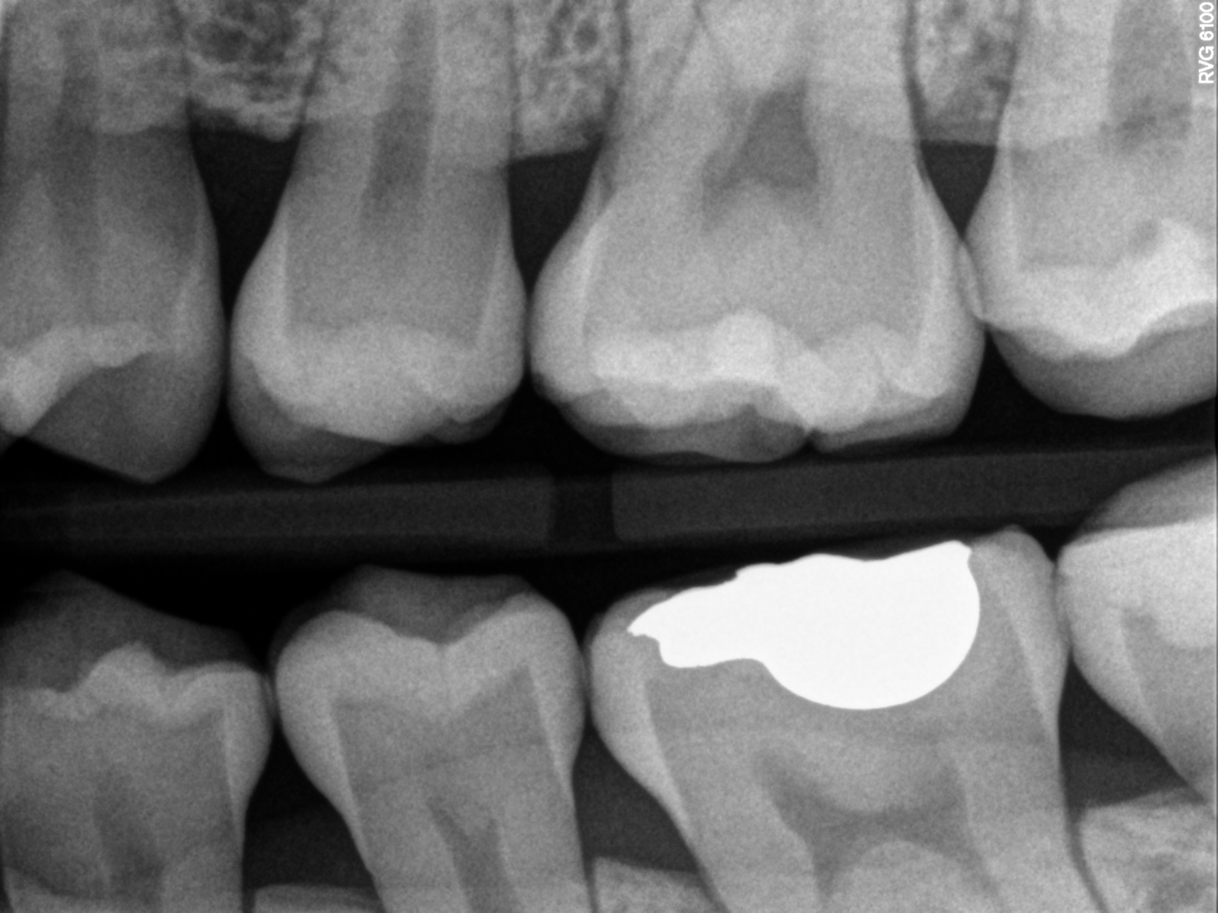


Image 5:


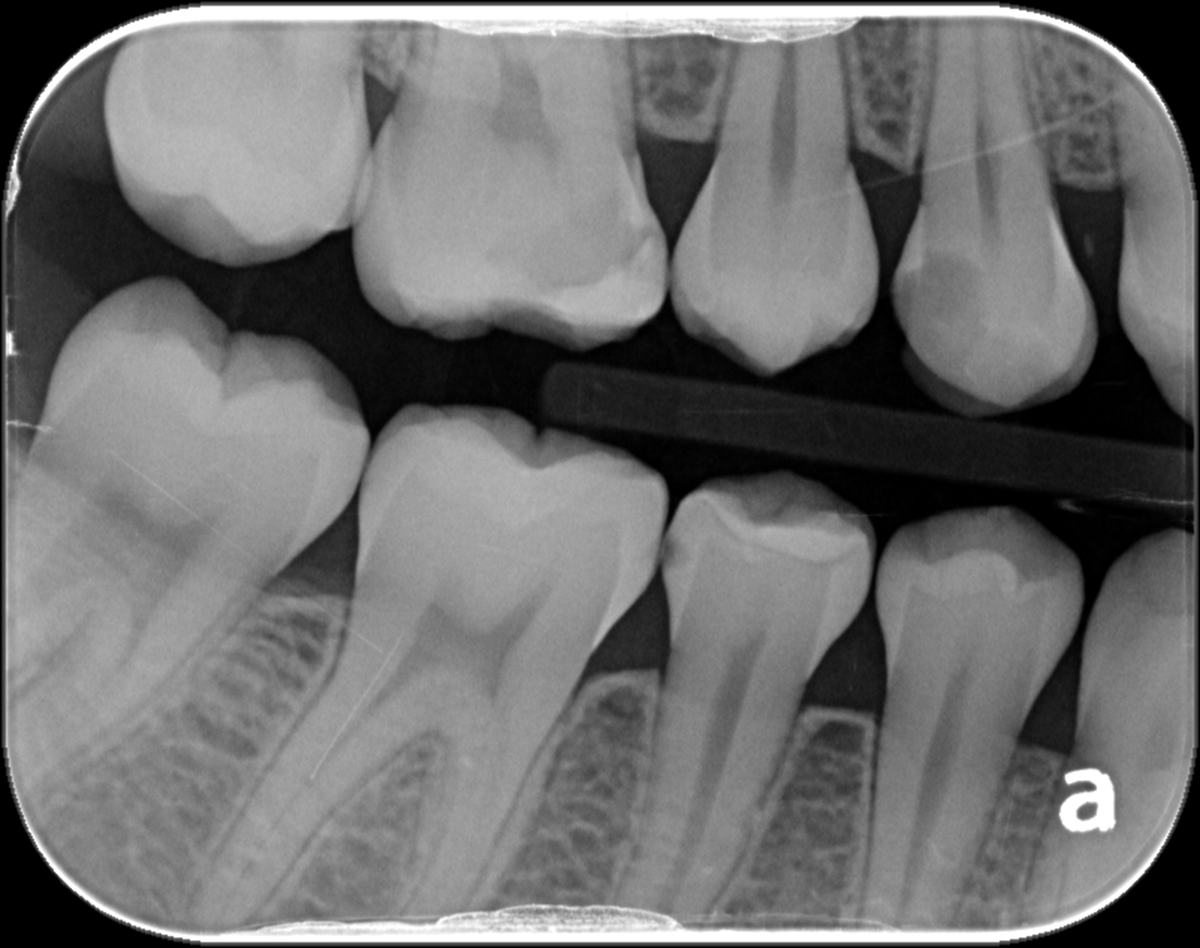


Image 6:


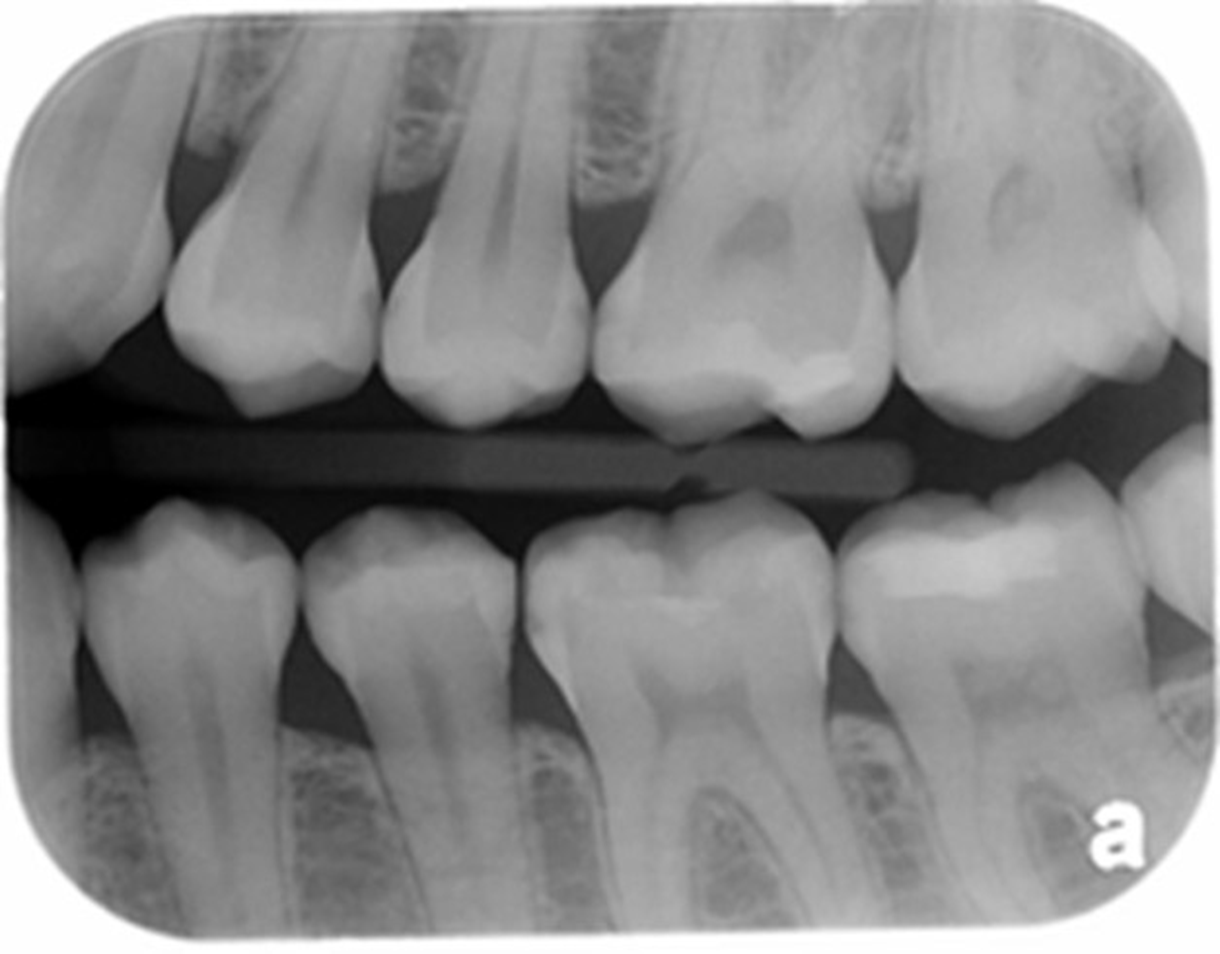


Image 7:


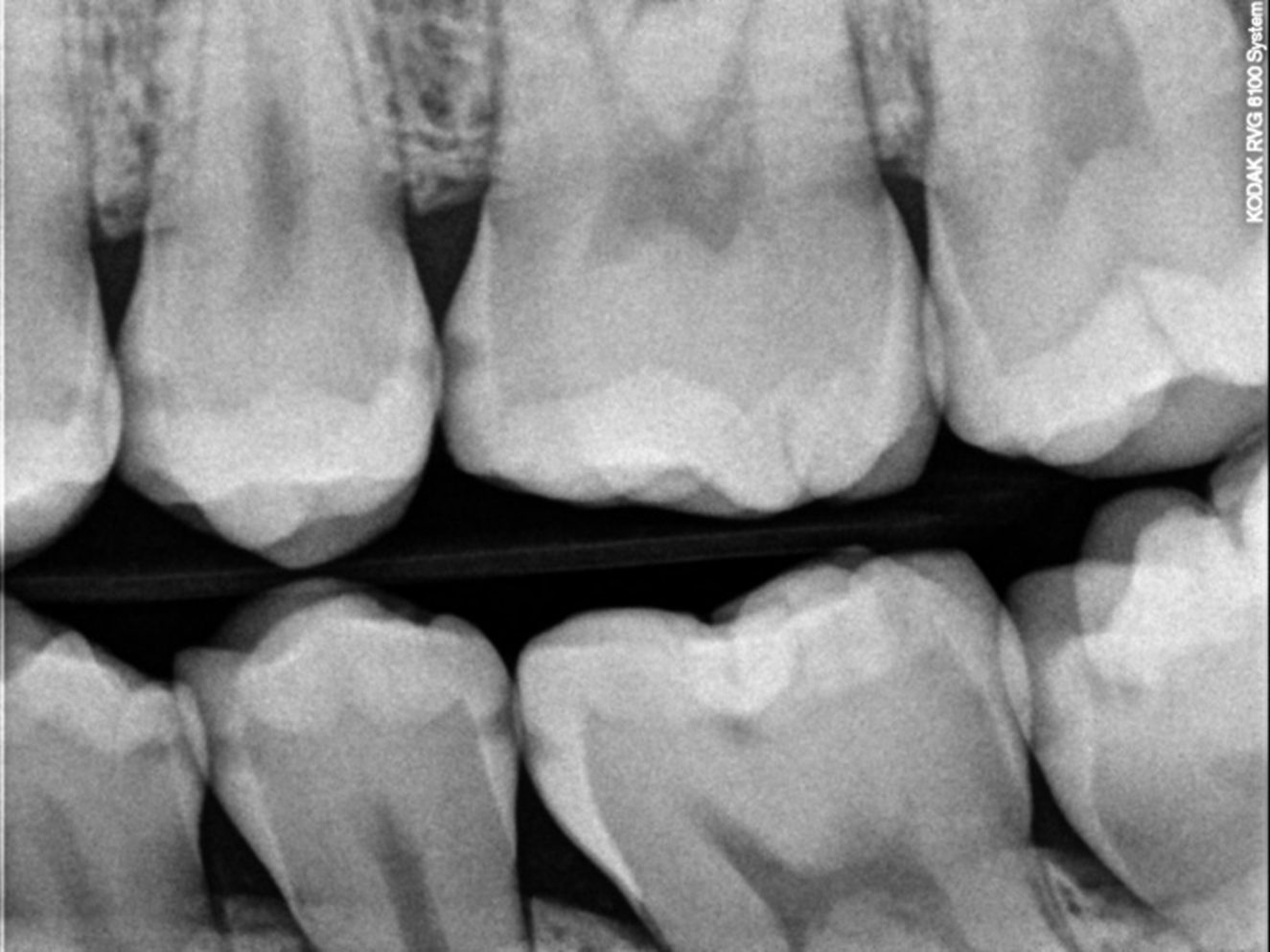


Image 8:
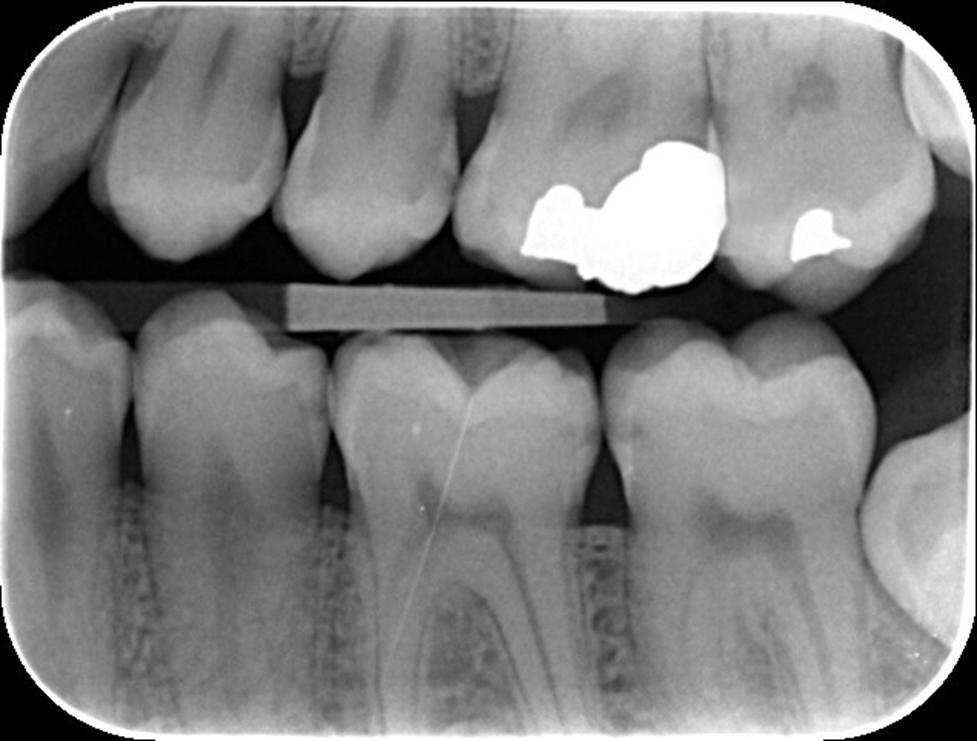


Image 9:


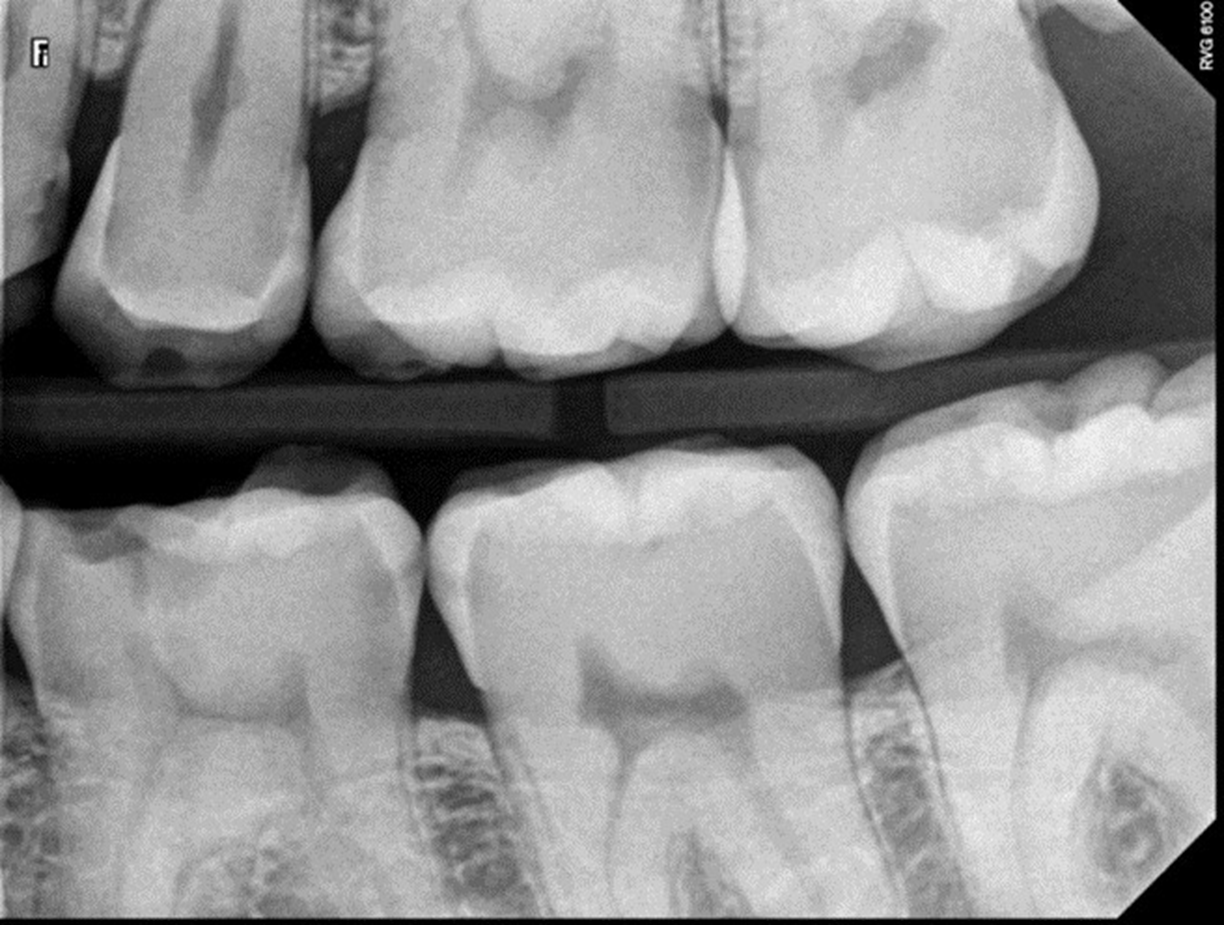


Image 10:


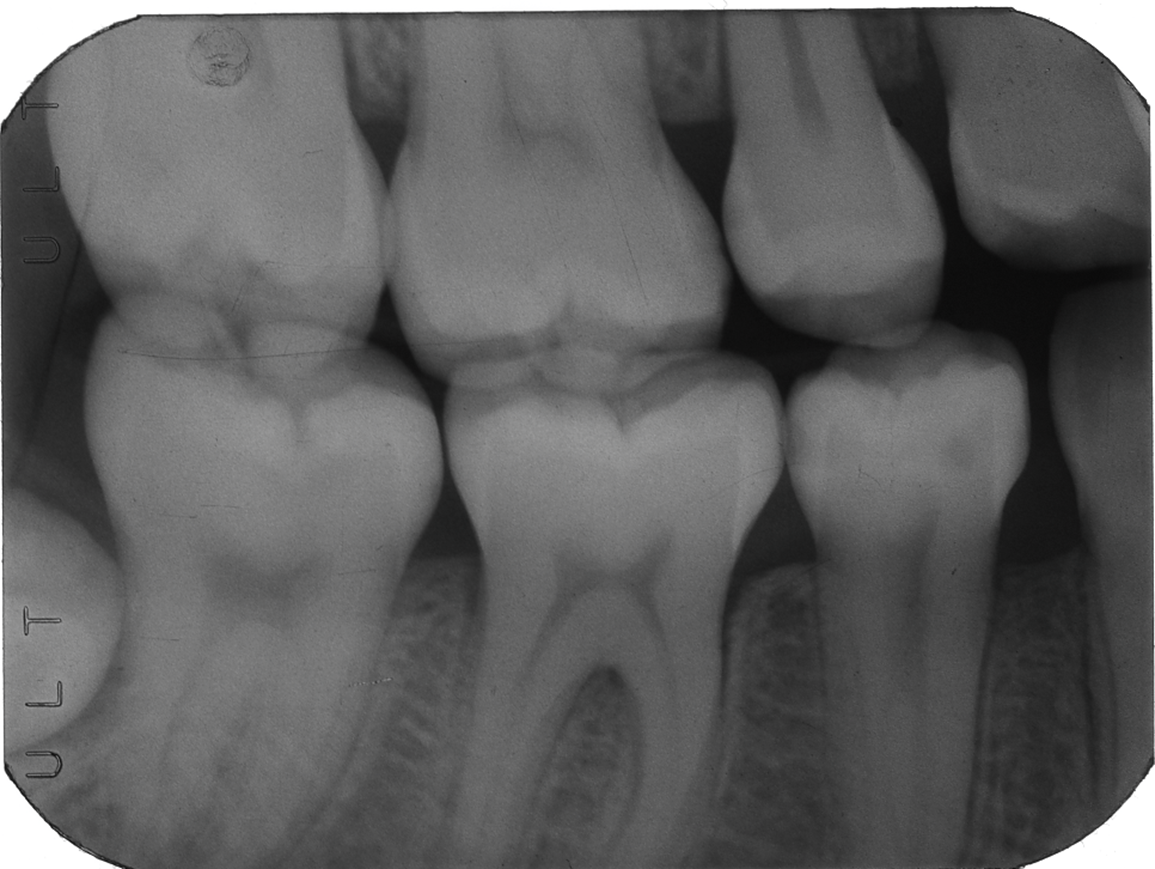

Supplement: twaf053_Supplementary_Data [file twaf053_supplementary_data.zip › Supplementary File 4.docx]
