# Supplementary material for: Machine learning algorithms enhance the accuracy of radiographic diagnosis of dental caries: a comparative study
Source: Dentomaxillofac Radiol. 2025 Jul 10;54(8):632–41. doi: 10.1093/dmfr/twaf053 (PMC12653770; doi:10.1093/dmfr/twaf053)
Supplement: twaf053_Supplementary_Data [file twaf053_supplementary_data.zip › Supplementary tables.docx]

| **Diagnostic Metrics** | **Formula** | **Interpretation** |
| --- | --- | --- |
| Sensitivity (recall) | TP / (TP + FN) | The proportion of actual positives was correctly identified. |
| Specificity | TN / (TN + FP) | The proportion of actual negatives was correctly identified. |
| Diagnostic Odds Ratio | (TP / FN) / (FP / TN) | Odds of a positive test result being correct versus incorrect. Values >1 indicate a discriminatory ability; values>20 indicate strong discrimination. |

Supplementary Table S1: Description of diagnostic metrics used in this study and their interpretation

| Gender | 27 females (53.8%  24 males (46.2%) |
| --- | --- |
| Age | Mean age 24.7 ±4.3 years  Age range 23-36 years |
| Duration image interpretation | Average 23.1 (±6.2) minutes  ML 24.4 (±7.7) minutes  Control 21.6 (±5.6) minutes  Checklist 23.3 (±5.1) minutes |

Supplementary Table S2: Participant demographics and image interpretation time.

**Control group (n=17)**

| Question | Response | Count (%) |
| --- | --- | --- |
| Would you consider using a checklist? | Yes | 13 (76.5) |
|  | No | 4(23.5) |
| If yes, reasons for using a checklist | Prevents missed lesions | 10(58.8) |
|  | Makes interpretation systematic | 7(41.2%) |
|  | Saves time | 3(17.6) |
| If no, the reasons for not using a checklist | Wastes time | 3(17.6) |
|  | Inefficient | 2(11.8) |
|  | No need | 2(11.8) |
| Would you consider using ML? | Yes | 15(88.2) |
|  | No | 1(5.9) |
| If yes, reasons for using ML | Saves time | 6(35.3) |

**ML group (n=18)**

| Question | Response | Count (%) |
| --- | --- | --- |
| When did you use the prompts? | Before viewing the radiograph | 6(33.3) |
|  | After viewing the radiograph for assistance | 5(27.8) |
|  | After viewing the radiograph to confirm | 2(11.1) |
|  | Checked, but no effect | 1(5.6) |
|  | Missing data | 4(22.2) |
| Reasons for using the prompts | Validate diagnosis | 13(72.2) |
|  | Second opinion | 10(55.6) |
|  | Saves time | 8(44.4) |
|  | Trust ML | 3(16.7) |
|  | Not confident | 2(11.1) |
| Was your caries diagnosis in agreement with ML prompts? | Yes | 9(50) |
|  | No | 5(27.8 |
|  | Missing | 4(22.2) |
| Changed diagnosis after using ML? | Yes | 5(27.8) |
|  | No | No data |
|  | Missing | 13 (72.2) |
| Did the ML affect your confidence? | Yes | 6(33.3) |
|  | Sometimes | 7(38.9) |
|  | Missing | 5(27.8) |

**Checklist group (n=17)**

| Question | Response | Count (%) |
| --- | --- | --- |
| When did you use the checklist | Before radiograph | 5(29.4) |
|  | After viewing the radiograph for assistance | 7(41.2) |
|  | After viewing the radiograph to confirm | 2(11.8) |
|  | Missing | 3(17.6) |
| Reasons for using the checklist | More confident | 10(58.8) |
|  | More accurate | 2(11.8) |
|  | Missing | 3(17.6) |
| Reasons for not using the checklist | No increase in accuracy | 2(11.8) |
|  | Missing | 15(88.2) |
| Did the checklists help? | Yes | 7(41.2) |
|  | No | 7(41.2) |
|  | Missing | 3(17.6) |
| Did the checklist affect your confidence? | Yes | 3(17.6) |
|  | No | 6 (35.3) |
|  | Missing | 8(47.1) |

Supplementary Table S3: Post-task questionnaire responses. This table summarises participants’ responses regarding their use of and attitudes toward cognitive aids during caries diagnosis. Participants could select multiple options for some questions, and percentages may exceed 100%.
