# Supplementary figures and images for: Machine learning algorithms enhance the accuracy of radiographic diagnosis of dental caries: a comparative study
Source: Dentomaxillofac Radiol. 2025 Jul 10;54(8):632–41. doi: 10.1093/dmfr/twaf053 (PMC12653770; doi:10.1093/dmfr/twaf053)

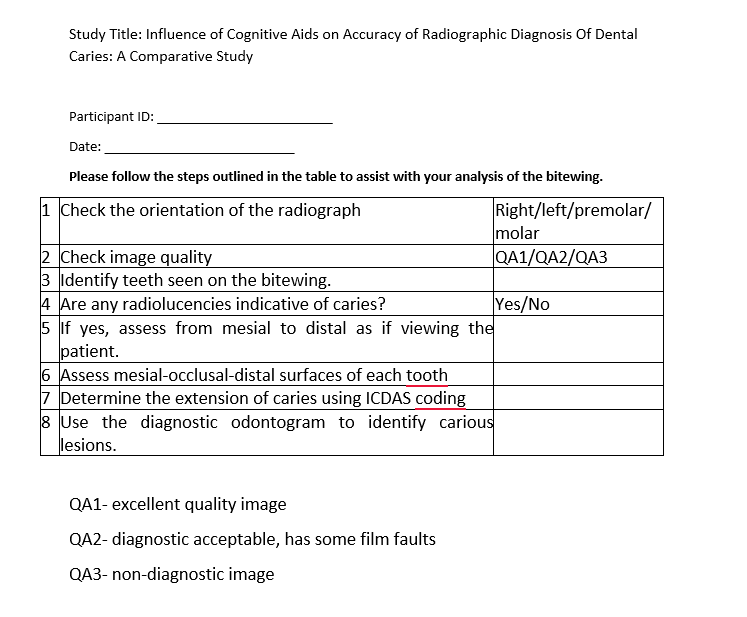

Supplement: twaf053_Supplementary_Data [file twaf053_supplementary_data.zip › Supplementary File 1.docx]
